# Effects of aerobic exercise and computer-based cognitive training on cognition, functional independence, quality of life, and salivary cortisol levels in older adults with mild cognitive impairment: a randomized trial

**DOI:** 10.3389/fnagi.2026.1776069

**Published:** 2026-03-16

**Authors:** Abdur Raheem Khan, Aafreen Aafreen, Ashfaque Khan, G. Shankar Ganesh, Monira I. Aldhahi, Mohammed M. Alshehri, Mohammad Abu Shaphe

**Affiliations:** 1 Department of Physiotherapy, Integral University, Lucknow, India; 2 Department of Health Rehabilitation Sciences, Faculty of Applied Medical Sciences, University of Tabuk, Tabuk, Saudi Arabia; 3 Department of Physiotherapy, Composite Regional Centre for Skill Development, Rehabilitation, and Empowerment of Persons with Disabilities, Lucknow, India; 4 Department of Rehabilitation Sciences, Princess Nourah bint Abdulrahman University, Riyadh, Saudi Arabia; 5 Physical Therapy Department, College of Nursing and Health Sciences, Jazan University, Jazan, Saudi Arabia

**Keywords:** aerobic training, age related, cognition, computer based cognitive training, exercises

## Abstract

**Background:**

Mild Cognitive Impairment (MCI) represents an intermediate stage between normal aging and dementia, characterized by measurable cognitive decline without significant loss of functional independence. Non-pharmacological interventions, particularly aerobic exercise and cognitive training, have demonstrated potential benefits; however, their combined effects and underlying neurobiological mechanisms remain inadequately explored.

**Objective:**

To investigate the effects of aerobic exercise combined with computer-based cognitive training on cognitive function, functional independence, health-related quality of life, and salivary cortisol levels in older adults with MCI.

**Methods:**

This assessor-blinded, two-arm randomized controlled trial included 60 older adults (60–85 years) diagnosed with MCI. Participants were randomized to either an intervention group receiving aerobic exercise plus computer-based cognitive training (*n* = 28) or an active comparison group receiving aerobic exercise with conventional cognitive training (*n* = 28). Interventions were delivered over 12 weeks, with assessments conducted at baseline, post-intervention (12 weeks), and follow-up (16 weeks). Primary and secondary outcomes included the Montreal Cognitive Assessment (MOCA), Barthel Index (BI), Short Form-12 (SF-12), and salivary cortisol levels. Data were analyzed using repeated measures ANOVA and Pearson correlation analysis.

**Results:**

Both groups demonstrated significant within-group improvements in cognitive function, functional independence, quality of life, and cortisol levels over time (*p* < 0.05). The intervention group showed significantly greater improvement in MOCA scores at 16-weeks (*p* < 0.05). Salivary cortisol levels differed significantly between groups at 12 weeks (*p* < 0.05). A significant negative correlation between cortisol levels and cognitive performance was observed at follow-up in both groups.

**Conclusion:**

Combining aerobic exercise with cognitive training improves clinical outcomes in older adults with MCI and may influence stress-related neurobiological pathways. This multimodal approach represents a promising, non-pharmacological strategy for mitigating age-related cognitive decline.

## 1 Introduction

Improvements in health care have decreased mortality rates in most countries, significantly increasing the average life expectancy to 65 years and above (Kinsella and Velkoff, 2001). Aging is a natural phenomenon-characterized by a gradual and permanent deterioration of physical function across all organ systems, leading older individuals toward a loss of mobility and independence, with many finally confronting institutionalization. Cognitive impairment is a challenge linked to the aging process and may serve as an indicator of Alzheimer's disease (Ritchie and Touchon, 2000) and dementia. Cognitive impairment is projected to affect 82 million people by 2030 and 152 million by 2050, with the global cost of dementia estimated at 1.1% of the global GDP in 2015 (Kuan et al., 2022). The financial strain on households is significant, with those containing a member with cognitive impairment requiring 48% more income to maintain their standard of living than similar households without such a member (Morris et al., 2021).

Mild Cognitive Impairment (MCI) is central to cognitive decline, positioning itself as a transitory phase between average age-related cognitive decline and early manifestations of dementia. Individuals with MCI exhibit cognitive disturbances, slight difficulty in complicated activities, and the capacity to carry out routine everyday functions (Etgen et al., 2011). The prevalence of MCI has been reported to be 19.7% globally, with a notable increase to 32.1% after 2019, particularly in hospital settings, with a worldwide cost estimate of 604 billion annually, which is expected to triple by 2050 (Song et al., 2023; Langa, 2015).

Over the years, interventions for MCI have taken multiple forms, including both pharmacological and non-pharmacological approaches, such as promoting independence in communication and activities of daily living, as well as maintaining a healthy lifestyle through regular physical activity and mental exercises (Ambigga et al., 2011). Research studies have demonstrated that aerobic exercise (AE) is useful in enhancing cognition in elderly individuals (Frisoni et al., 2023; Tsai et al., 2019; Yu et al., 2021). One of the primary benefits of AE is its ability to preserve and improve neurocognitive function across the lifespan, potentially by modifying stress-related pathways involving the hypothalamic-pituitary-adrenal (HPA) axis and autonomic nervous system, as indicated by changes in biomarkers, such as cortisol and salivary α-amylase (Anacker et al., 2016). Cortisol levels can be quantified via blood or saliva samples (Laudat et al., 1988). Research indicates that both extremely elevated and diminished cortisol levels can negatively impact cognitive performance (Drogos et al., 2019; Lee et al., 2022). Growing data shows a strong relationship between elevated cortisol levels and faster progression of cognitive deterioration (Cuadrado-Tejedor et al., 2012). AE also enhances cerebral microcirculation, particularly in deep cortical layers and subcortical white matter, which are vulnerable to aging-induced decline. Furthermore, AE contributes to the construction of brain and cognitive reserves, particularly affecting memory and executive function, through structural and functional changes in the fronto-hippocampal axis (Di Liegro et al., 2019). Another work considers AE as a type of cognitive training (Rao et al., 2018).

Cognitive therapies have demonstrated efficacy as a potential therapy choice for patients with MCI (Hwang et al., 2012). The impact of these therapies is posited to arise from the augmentation of cognitive reserve, which bolsters resilience against neurodegeneration (Stern et al., 2020) and enhances neuroplasticity (Chapman et al., 2015). Nonetheless, prior research has not consistently demonstrated the efficacy of cognitive intervention in MCI, with statistically significant improvements observed in just about fifty percent of the published trials (Jean et al., 2010; Kurz et al., 2009). Moreover, many prior studies lacked individualization, had small sample sizes, and did not undertake follow-up evaluations (Belleville et al., 2006; Talassi et al., 2007; Wenisch et al., 2007), and these programs should be tailored to the patient's cultural and social context.

Recent developments in computing technology have enabled researchers to conduct cognitive training within this population. In addition to improved accessibility and cost-efficiency, technology-based interventions offer immersive and complete adaptive responses tailored to individual performance. Significantly, computer-based interventions have demonstrated superior outcomes relative to conventional cognitive training and rehabilitation programs in sustaining physical activity and mental health and enhancing cognitive function and quality of life (Lau et al., 2022). Although technology-based cognitive training has shown substantial benefits across areas with moderate to large effect sizes, the transferability of these training gains varies depending on the intervention and delivery mechanism (Ge et al., 2018). Further, though different treatments have shown benefit in reducing cortisol levels, the relationship between clinical improvement in cognitive function and a reduction in cortisol levels has not been examined in a sample that receives conventional and computerized cognitive training. Moreover, the extent to which cortisol influences cognitive performance across various interventions remains ambiguous, as is the potential for these interventions to elicit divergent cortisol response patterns (Pontifex et al., 2019). Consequently, additional research is necessary to determine which intervention would most effectively improve clinical outcomes. The current study sought to examine trends in salivary cortisol levels among patients with MCI and to compare the impacts of aerobic and cognitive interventions on various clinical outcomes, including the Montreal Cognitive Assessment (MOCA), Barthel Index (BI), Short Form Survey-12 (SF-12), and salivary cortisol levels. The secondary purpose is to assess the correlations between cognitive functioning and salivary cortisol concentrations after the intervention.

## 2 Methodology

### 2.1 Study population and design

This study focused on a cohort of elderly individuals with MCI. The structure of the study was a meticulously designed proof-of-concept, two-arm parallel, randomized controlled trial, conforming to the stringent CONSORT guidelines, ensuring the study's reliability and completeness. Informed consent was obtained from all participants, who provided a detailed account of the study's aim, expected procedures, potential risks, and benefits.

Sixty individuals were strategically recruited based on the specific inclusion and exclusion criteria. The participants, both male and female, aged 60–85, were recruited from Jazan University, Jazan, or the surrounding community and met the criteria for MCI (Albert et al., 2013). Participants possessing MoCA scores between 22 and 26 (Segal-Gidan, 2013) who can comprehend basic English/Arabic and adhere to verbal instructions were included in the study. Individuals diagnosed with Alzheimer's disease, Lewy body disease, cerebrovascular accidents, Huntington's disease, multiple sclerosis, amyotrophic lateral sclerosis, frontotemporal dementia, and vascular dementia were excluded from the study. Any serious or unstable medical condition that may hinder the patient from fulfilling all study criteria (e.g., unstable or severe asthma, cardiovascular illness, or severe hepatic or renal disease); those taking medications detrimental to cognition, such as benzodiazepines; users of antipsychotics and cholinesterase inhibitors; individuals with current or recent alcohol or substance use disorders; those exhibiting behavioral changes like depression, anxiety, or stress; patients with systemic diseases potentially impairing cognition, such as hypothyroidism or deficiencies in folic acid or vitamin B12, venereal diseases or viral infections like syphilis or HIV; individuals with vision and speech impairments (aphasia); those experiencing musculoskeletal pain; individuals with insufficient sleep (less than 8 hours), oral ulcers, oral wounds, or dental carie; and those with nutritional deficiencies, such as thiamine (Eleni et al., 2017), were excluded. This trial was registered at ClinicalTrials.gov (NCT06226103) and was approved by the Institutional Ethical Committee of Jazan University, Jazan, Saudi Arabia (REC-45/06/919).

### 2.2 Sampling method

Participants were assigned to groups using a computer-generated random number sequence in blocks to ensure an unbiased and non-discriminatory selection process. Group (arm) allocation was not disclosed to the participants, and they were asked not to discuss the intervention during the training sessions. The outcome assessors were blinded to the allocation and were not involved in the interventions.

### 2.3 Power and sample size

The study used G. Power 3.15 software (Franz F, Universität Kiel, Kiel, Germany) to determine the number of participants needed for the experiment. The calculation was based on net improvement, and it was found that 30 subjects per group, including 10% dropouts, were required. An effect size of 0.30, an alpha level of 0.05, and a power (1-beta) of 0.80 were taken into consideration during the calculation (Bradley and Brand, 2013). With the recruitment of 30 participants, we can ascertain that our study achieved 80% power at the 5% significance level to identify an effect size of 0.30, while considering a dropout rate of 10% observed after 16 weeks.

### 2.4 Outcome measures

#### 2.4.1 Montreal cognitive assessment (MOCA)

This tool evaluates seven domains of cognition, including memory recall, spatial and executive abilities, attention span, concentration levels, and working memory capacity. The highest attainable score is 30 points, and a score of 25 or lower signifies cognitive impairment. MOCA is a reliable and valid measure for assessing cognitive performance across various domains (Nijsse et al., 2017).

#### 2.4.2 Barthel index (BI)

This index quantitatively measures an individual's capability to execute ten fundamental self-care and mobility activities, which are integral to one's functional independence. The scale assesses an individual's capacity to execute activities of daily living and mobility. The highest attainable score is 100, with a higher score signifying enhanced functional independence. BI is well-established in the literature as a reliable and valid instrument for measuring daily functioning (Hong et al., 2016).

#### 2.4.3 Short form survey-12 (SF-12)

The SF-12 is a reliable and valid tool to evaluate health-related quality of life. This instrument comprises 12 enquiries regarding constraints in everyday activities attributable to health, discomfort, energy levels, and psychological distress. The scores are categorized into physical and mental components, with higher values indicating a superior quality of life (Jakobsson, 2007).

#### 2.4.4 Measurement of salivary cortisol levels

Saliva has gained heightened attention as a biospecimen due to its cost-effectiveness and little invasiveness (Dickerson and Kemeny, 2004), and salivary analyses may identify stress-related biochemical pathways in elderly adults with neurocognitive problems (Navazech, 1993). The salivary samples were obtained in the mid-morning. Participants were prohibited from ingesting stimulants within 3 h before the test, and the collection team refrained from obtaining samples within 1 h following a substantial meal. The patient was directed to rinse the mouth with water 10 min before saliva collection. The samples (75 μl) were obtained in a head-forward posture in a tube under stringent aseptic conditions. Contaminated samples were discarded and excluded from examination. The collection team stored the samples in the freezer until they were dispatched for examination. The enzyme-linked immunosorbent assay was employed to measure salivary cortisol concentrations in accordance with the manufacturer's specifications. The gathered samples were centrifuged within 2 h to isolate saliva, and the supernatant was examined using an enzyme-linked immunoassay detector.

### 2.5 Procedure

Prior to the random allocation of individuals to treatment groups, independent evaluators not affiliated with the study gathered critical demographic information, including age, body weight, and height, and computed the Body Mass Index and outcome variables, such as the MOCA, BI, salivary cortisol level, and SF-12 at baseline (day 0), at the conclusion of treatment (after 12 weeks), and after a four-week follow-up (after 16 weeks). Four experienced physiotherapists participated in a training session and implemented the interventions for a duration of 12 weeks. They refrained from interacting with individuals beyond data collection and remained unaware of the group allocations to preserve their blindness. Prior to the experiment, participants were informed about the study's purpose, the variables to be gathered, and the potential advantages and risks involved. The participants were told they could leave the study at any time. All the participants granted informed consent.

#### 2.5.1 Arm 1 (intervention group A)

Participants engaged in structured AE three times a week for a span of 12 weeks. The participants underwent cognitive training tailored based on individual assessment scores three times a week sequentially.

The AE program involved gradually increasing the intensity and duration of brisk walks. Each session commenced with a warm-up phase consisting of 5–10 min of brisk walking, followed by 5 min of dynamic stretching targeting the trunk and lower limb muscles. Participants donned heart rate monitors to record their heart rates. The participants concluded each session with a 5- to 10-min cool-down period consisting of low-intensity walking, followed by static stretching of lower-limb muscles. In the first week, participants were asked to walk briskly for 30 min, 3 days a week, at a self-selected pace. The intensity was increased during the second week to an intensity of 9–10 on the Borg Rating of Perceived Exertion Scale (BRPES; Borg et al., 1987), and the duration was extended to 45 min while maintaining the same intensity and frequency (3 days per week). For the next 10 weeks, participants continued to walk for 45 min, 3 days per week, but the intensity was gradually increased to 12–14 on the BRPES, which is considered moderate to high effort. The entire session was monitored by four physiotherapists with postgraduate qualifications and more than 5 years of clinical experience to ensure the participants' safety.

#### 2.5.1 Computerized cognitive training

BrainHQ (Posit Science Inc., San Francisco, CA, USA) is a computer application utilizing game-based methods to train diverse cognitive skills by boosting neuroplasticity and showing substantial effects on older individuals living in the community (Zelinski et al., 2011). This study utilized touchscreen tablets to engage participants in games such as Double Decision, Hawk Eye, Divided Attention, etc., that targeted cognitive skills such as visuospatial processing, attention, memory, language, and logical reasoning. Participants were directed to utilize their dominant or preferred hand for operating the touchscreen tablets. The participants engaged in two to three games for a duration of 90 min, with the complexity of the tasks tailored to each individual's proficiency and capacity. The task became increasingly difficult as a person progressed. The intervention comprised 10 min of warm-up, 70 min of cognitive training, and 10 min of cool-down. Four postgraduate-qualified occupational therapists with more than 5 years of clinical geriatric experience administered and monitored these sessions. The performance of each participant was documented in the daily log. The participants received one or two games for practice during the training session.

#### 2.5.2 Arm 2 (active comparison group-B)

Participants in this group engaged in the AE program administered to intervention group A with an identical dosage for a duration of 12 weeks. The participants were taught conventional cognitive training and asked to perform these exercises at home for a duration of 90 minutes three times a week after the AE. The cognitive training program encompassed mnemonic memory retrieval tasks, attention enhancement activities including the identification of target numbers/words, visual pattern scanning, task switching, and the application of metacognitive strategies such as Go-Plan-Do-Check to accomplish everyday tasks (Jolles and Crone, 2012). If patients declined to participate in the cognitive program following AE, the training would be deferred until the following day. An active control group mitigates biases by regulating positive perceptions of the intervention and ensuring participant blindness. Participants were directed to follow the interventions according to the exercise protocol while maintaining their regular levels of physical activity on the remaining days.

#### 2.5.3 Home training program (4 weeks)

The home exercise programmes were recommended to ascertain whether the advantages acquired during the intervention are sustained after the cessation of official supervision of the organized programme. Additionally, these home activities are necessary to assess the sustainability of behavioral modifications, including a long-term commitment to exercise. Moreover, home-based exercise is cost-effective and simple to execute over extended periods.

All the participants (Arm 1 and 2) were advised to continue their aerobic activity at home. AE involves brisk walking on a level surface to attain the desired intensity (the level at which one can talk but not sing) for a duration of 30 min (Yang, 2019). Patients and their family carers were provided with a diary to record the exercises performed within designated timeframes each day and on specific days of the week. The patients were directed to engage in AE and perform cognitive exercises (60 min a day) three times weekly. Tablet usage for cognitive training at home was prohibited during the home-exercise session. Researchers conducted biweekly phone follow-ups to ensure participants completed their home exercises.

### 2.6 Statistical analysis

Descriptive statistics were applied to examine the primary attributes, such as the mean, standard deviation, and percentages. Data normality was assessed using the Shapiro-Wilk test. The primary analysis employed linear mixed-effects modeling for repeated measures across time, utilizing the MOCA score as the dependent variable and time, group, and the time-by-group interaction as independent variables. Temporal changes within each group and the intergroup differences in the MOCA score, together with the associated 95% confidence intervals, were documented. A similar analysis was performed for BI, SF12, and salivary cortisol levels. The significance criterion for all statistical tests was established at p < 0.05. An intention-to-treat analysis was conducted with all participants in their designated groups to validate randomization and mitigate potential dropout effects. In instances of dropout, values from the prior time point were substituted to ensure the robustness of the findings. Post-hoc analyses were performed using Bonferroni corrections to determine the specific time points at which significant differences were observed between the groups. Pearson correlation analysis was employed to ascertain the association between cognitive functioning and salivary cortisol levels. The significance level was established at *p* < 0.05. The data were analyzed using SPSS version 25.0.

## 3 Results

**Figure 1:**
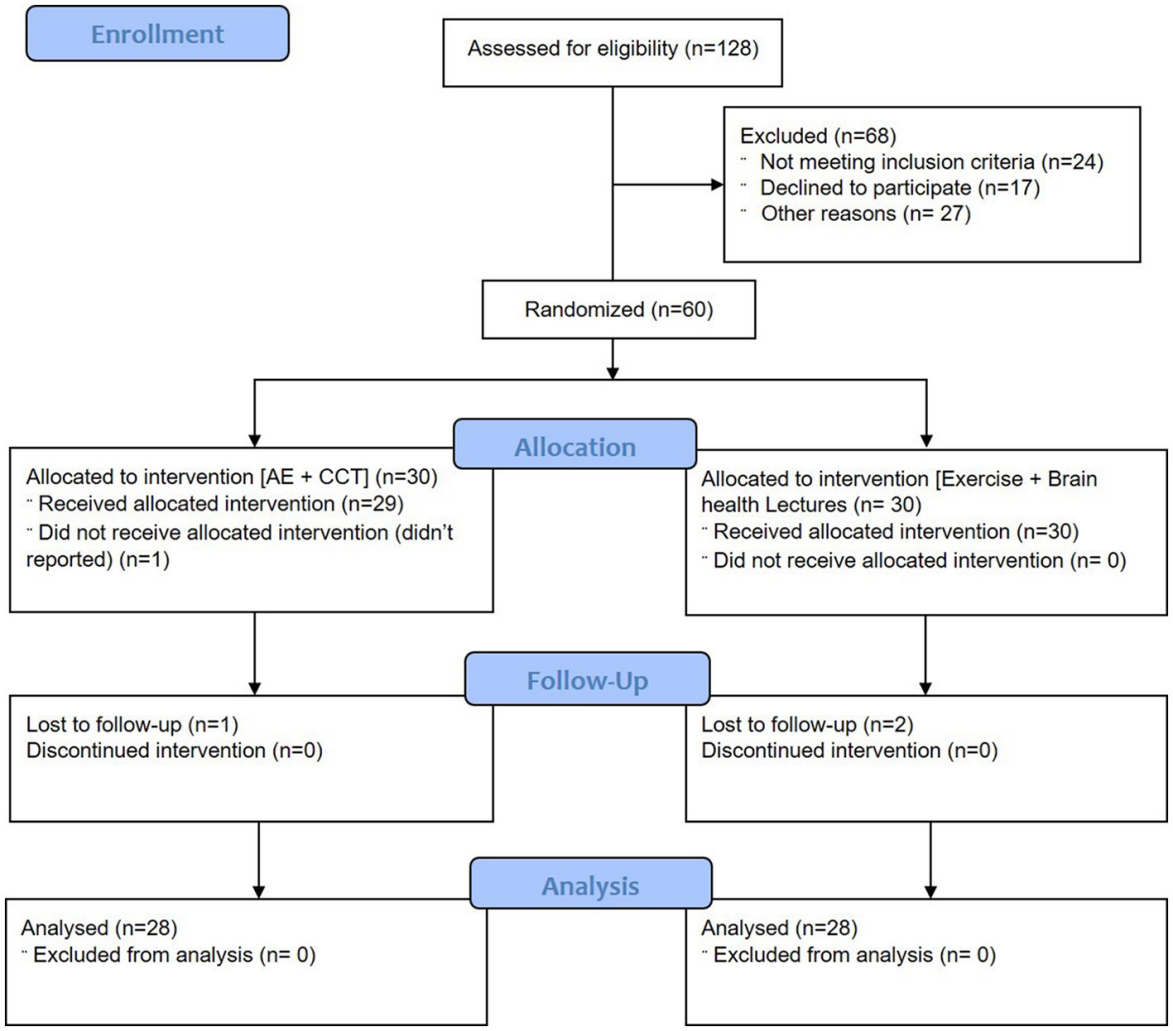
CONSORT diagram showing the number of patients in the treatment groups.

A total of 60 participants were assigned out of 128 participants enrolled from the Department of Physiotherapy & University Hospital, Jazan University, and nearby communities, from June 16, 2022, to August 25, 2023. Four participants dropped out for different reasons (Figure 1). Of the 56 participants who completed the study, 28 received intervention in the form of computer-based cognitive training combined with aerobic training (Group A, Interventional), and 28 received AE with conventional exercises (Group B, Control) presented in Figure 1.

### 3.1 Demographic characteristics

The mean age of participants was similar between the intervention group (70.80 ± 8.10 years) and the control group (71.36 ± 6.86 years). The distribution of gender was also comparable between groups, with females comprising 40% (*n* = 11) of the intervention group and 35% (*n* = 10) of the control group. The mean body mass index (BMI) for the intervention group was 25.40 ± 3.72 kg/m^2^ compared to 24.89 ± 2.46 kg/m^2^ for the control group. All outcome measures (MOCA, BI, and SF-12) were normally distributed, and there were no significant demographic differences between the intervention and control groups at baseline. The patients' demographic characteristics are presented in Table 1.

**Table 1:** Demographic characteristics of participants.

**Characteristic**	**Group A (Intervention) *N* = 28**	**Group B (control) *N* = 28**	**Chi-square/*t*-value**	***p*-value**
Age (mean SD)	70.80± 8.10	71.36± 6.86	0.28	0.78
Female (%)	11 (40%)	10 (35%)	0.076^*^	0.78^*^
Male (%)	17 (60%)	18 (65%)		
Weight (mean SD kg)	65.62 ± 10.13	63.93 ± 7.14	0.72	0.47
Height (mean SD m)	1.61 ± 0.09	1.60 ± 0.09	0.42	0.68
BMI (mean SD kg/m^2^)	25.40 ± 3.72	24.89 ± 2.46	0.61	0.55

^*^Chi-square test.

### 3.2 Outcome measures

Table 2 presents the cognitive functioning, quality of life, activities of daily living, and salivary cortisol scores among patients in different groups.

**Table 2:** Comparison of clinical outcomes between two study groups.

**Outcome by group**	**Summary at different time point a mean (SD)**			**From baseline to 12 weeks mean (95% CI)**		**From baseline to 16 weeks mean (95% CI)**	
	**Baseline**	**12-weeks**	**16-weeks**	**Within-group change**	**Between-group difference in change**	**Within-group change**	**Between-group difference in change**
**MOCA**							
Group 1	23.2 (1.9)	24.7 (2.2)	25.0 (2.4)	1.6 (0.8,2.3)^*^	0.8 (−0.1, 1.8)	1.9 (1.0, 2.8)^*^	1.2 (−1.0, 2.4)^*^
Group 2	22.9 (2.1)	23.6 (1.8)	24.7 (2.4)	0.7 (0.0, 1.4)^*^		1.7 (0.8, 2.6)^*^	
**BI**							
Group 1	84 (29)	92 (25)	98(20)	8.7 (0.2, 17.2)^*^	7.4 (4.3, 19.0)	15 (6, 24)^*^	10 (2, 23)
Group 2	86 (23)	94 (20)	99 (22)	5.3 (3.7,9.4)^*^		6.9 (3.8, 13.6)^*^	
**SF-12**							
**Group 1**	40.0 (8.5)	47.2 (8.2)	48.0 (9.0**)**	5.2 (0.1,10.4)^*^	2.4 (−4.6, 9.4)	6.1 (1.1, 11.2)^*^	5.6 (−1.5, 12.7)
**Group 2**	39.8 (8.6)	45.0 (8.7)	46.2 (8.1)	2.8 (−2.0, 7.7)^*^		0.5 (−4.5, 5.5)^*^	
**Salivary cortisol**							
Group 1	18.4 (4.7)	16.1 (4.3)	14.7 (4.2)	−3.9 (−4.9, 0.4)^*^	1.7 (−1.3, 4.1)^*^	−2.9 (−4.2, 0.1)^*^	1.2 (−1.9, 4.2)
Group 2	17.6 (4.6)	14.1 (5.2)	13.9 (3.8)	−3.4 (−5.2, −1.5)^*^		−3.2 (−5.4, −1.1)^*^	

^*^p < 0.05.

MOCA, Montreal cognitive assessment; BI, Barthel index; SF-12, short form health survey 12.

#### 3.2.1 Within-group comparison of patient outcomes

Patients in the intervention group exhibited significant improvements at 12 weeks in cognitive function (mean increase in MoCA 1.6; 95% CI 0.8, 2.3; *p* < 0.001), activities of daily living (mean changes in BI 8.7; 95% CI −0.2, 17.2; *p* < 0.05), quality of life (mean changes in SF-12 5.2; 95% CI −0.1, 10.4), and salivary cortisol levels (mean change −3.9; 95% CI −4.9, 0.4; *p* < 0.05). These improvements were reflected after 16 weeks.

Patients in the control group exhibited a significant enhancement in cognitive function at 12 weeks, with a mean increase in MoCA of 0.7 (95% CI 0.0, 1.4; *p* < 0.05), a BI score change of 5.3 (95% CI 3.7, 9.4; *p* < 0.05), an improvement in quality of life with a mean change of 2.8 (95% CI −2.0, 7.7; *p* < 0.05), and a reduction in salivary cortisol with a mean score change of −3.4 (95% CI −5.2, −1.5; *p* < 0.01). An analogous enhancement was noted after 16 weeks, as well (Table 2).

#### 3.2.2 Between-group comparison of patient outcomes

Patients in the experimental group exhibited a more significant enhancement in cognitive function at 16 weeks compared to those in the control group (difference in MOCA changes 0.2, 95% CI −1.0, 1.4; *p* < 0.05) and in salivary cortisol levels at 12 weeks (mean difference 1.7, 95% CI −1.3, 4.1; *p* < 0.05). No differences were seen between the groups in other outcome measures at any time point (Table 2).

#### 3.2.3 Correlation between cortisol and cognitive function

The association between cortisol levels and cognitive functioning was assessed using Pearson's correlation coefficient after interventions. Results indicated that cortisol levels exhibited a negative, albeit non-significant, association with MOCA at the 12th week (*r* = −0.30, *p* = 0.087) for the experimental group, while a significant negative correlation was observed in the arm 2 participants (*r* = −0.45, *p* = 0.008). Cortisol had a substantial negative correlation in the 16th week with both the experimental group (*r* = −0.43, *p* = 0.012) and the control group (*r* = −0.35, *p* = 0.019).

## 4 Discussion

This study investigated the synergistic benefits of integrated computer-based cognitive training and AE in older persons with MCI. The principal findings from our study indicated an enhancement in the MOCA, BI, SF-12, and salivary cortisol levels among participants in both groups from pre- to post-measurement at all time points. Nonetheless, cortisol and MOCA results demonstrated enhancements across the groups at the 12th and 16th weeks, respectively. A statistically significant association between cortisol levels and MOCA scores was observed exclusively in the control group at the 12th week and at the 16th week in both groups.

Our study's participants averaged baseline MOCA scores of 23, which is consistent with other research that suggests MCI is characterized by scores below 26 (Nasreddine et al., 2005). In accordance with studies indicating that a minimal clinically relevant difference for MOCA ranges from 1 to 2 points (Lindvall et al., 2024), our findings demonstrated that both therapies effectively enhanced cognitive performance. While AE may primarily enhance brain health through physiological changes, cognitive training may bolster neural efficiency and cognitive reserves, leading to more substantial improvements when combined. This effect is frequently ascribed to augmented cerebral blood flow, improved neurogenesis, and the overexpression of neurotrophic factors, including brain-derived neurotrophic factor, which is crucial for neuronal survival, growth, and differentiation (Voss et al., 2013). The efficacy of AE is ambiguous, as a meta-analysis by Han et al. (2023) indicated enhanced cognitive functions, whereas the meta-analysis by Lin et al. (2023) found that walking had no significant impact on cognitive function in MCI patients.

Lee et al. found that cognitive training in older adults not only improved cognitive performance but also maintained brain structure, such as caudate volume and white matter integrity, which are crucial for cognitive control (Lee et al., 2024). Concurrent with these findings, computer-based cognitive training programs such as BrainHQ have been designed to challenge and stimulate various cognitive domains, which can lead to neuroplastic changes and the consequent enhancement of cognitive abilities (Lampit et al., 2014). The hypothesis posits that computerized training outperforms conventional cognitive training due to its ability to automatically adapt exercises to align with individual cognitive requirements (Lustig et al., 2009). Karssemeijer et al. (2017) conducted a meta-analysis revealing that integrated cognitive and physical exercise training markedly enhanced cognition scores relative to standard care therapies in this patient cohort. The notable enhancement after 16 weeks, however, may be attributed to the dosage necessary for eliciting neuroplastic change. Prior studies have demonstrated inconsistency in this regard; while one study indicated no effect on cognitive performance using the MoCA assessment after a 3-month intervention (Barnes et al., 2013), other trials have reported improvements with longer durations (Lazarou et al., 2017). Likewise, we hypothesize that the prescribed dosage of exercises might have become effective at the 16th week, as the effects may vary with differing exercise intensities (Amjad et al., 2019; Chang et al., 2021).

Scores of 50 and higher on the SF-12 signify a health-related quality of life that is better than average; nevertheless, the individuals in this sample exhibited lower scores, reflecting a diminished quality of life. A variation of three to five points in SF-12 often indicates a clinically significant enhancement in overall well-being and functional outcomes. Memory deficits are independently linked to deterioration in quality of life. The cognitive therapies provided could have enhanced the improvements in verbal, visual, and working memory and, thereby, activities of daily living and quality of life (Chan et al., 2024). The lack of statistical significance between the groups suggests that both therapies have comparable success in improving quality of life. This non-significance may be attributed to the lack of interventions targeting the psychosocial components of health, which are significant concerns among older individuals (Cornwell and Waite, 2009).

The mean baseline Barthel Index in our cohort was 85, indicating notable dependency in the execution of daily living activities. Although individuals exhibit significant autonomy in basic self-care, impairments in complex instrumental activities yield unexpectedly low ratings. Such outcomes may be related to confounding variables, such as the number of comorbidities. The ceiling effect may account for the absence of intergroup differences, despite the groups' ongoing improvement over the intervention period (Lim et al., 2015). Further, it's probable that cognitive training may transfer skills and performance solely to the trained tasks, rather than to closely related activities or other performance (Simons et al., 2016). Findings from a review indicated that whereas training over 36 h enhances visual and verbal episodic memory, no enhancement is noted in working memory (Chan et al., 2024). Another review revealed that cognitive training fails to implement suitable interventions; hence, it does not address the problem of generalization in everyday life (Belleville, 2008).

Elevated cortisol levels correlate with reduced brain volume (Geerlings et al., 2015), cognitive function (Cox et al., 2015), and performance (Beluche et al., 2010) and may hinder hippocampus-dependent learning and memory in humans (Sroykham, 2019). Various subtypes of MCI have been correlated with salivary cortisol; while elevated salivary cortisol levels are observed in non-amnestic MCI and multidomain MCI, amnestic MCI exhibits normal levels. While a negative link between cognitive scores and cortisol levels is evident, some studies indicate that salivary cortisol levels are associated with cognitive decline solely among those at risk for Alzheimer's disease (specifically, apolipoprotein E4 carriers; Gerritsen et al., 2011). Moreover, research suggests cortisol levels are correlated with MCI (Wolf et al., 2002), and low scores of cortisol are not correlated with any brain volume metrics (Echouffo-Tcheugui et al., 2018). The intricate diurnal pattern of cortisol release further hinders the interpretation of salivary cortisol levels.

Research indicates that AE (Smyth et al., 2019) and cognitive interventions (Da Silva et al., 2021) diminish cortisol levels and sustain cognitive performance by alleviating allostatic load and facilitating HPA axis modulation. The absence of correlation between cortisol levels and cognition in the experimental group at the 12th week may be ascribed to the causal dose-response relationship between the intensity of AE and HPA-axis cortisol reactivity (Caplin et al., 2021). Another review (Li and Huang, 2025) determined that an inverted U-shaped correlation exists between exercise intensity and cortisol lowering. The same review indicated that extended intervention duration forecasted more significant decreases in cortisol levels, which may explain the substantial decrease in cortisol levels in the experimental group when participants engaged in home activities.

Further, senior individuals may exhibit diminished interest in gaming. Despite both groups receiving comparable dosages of AE, the enhanced results of computerized cognitive training may be ascribed to its engaging and interactive characteristics (Cruz Gonzalez and Fong, 2018). Computerized cognitive training may elicit a stress-like physiological reaction, which is shaped by an individual's biological profile, gaming history, and the nature of the game content (Wang et al., 2023). The intense acute stress from gaming may have activated the sympathetic-adrenal medulla, resulting in the production of catecholamines (George et al., 2010). Prior research indicates that the cortisol reactivity of individuals under stress may vary from that of healthy individuals due to many external factors (Gerber et al., 2020). Significantly, a study found no correlation between cortisol levels and cognitive deterioration (Comijs et al., 2010). In our study, the majority of participants favored remaining at home over attending treatment sessions, perhaps alleviating the stress stemming from the psychological and physiological demands of regular attendance over the follow-up period. Given the moderate relationship between cortisol and cognitive functions after exercise interventions, future research should assess the impact of β-amyloid levels, as elevated β-amyloid is linked to diminished performance on neuropsychological assessments in cognitively impaired individuals (Luis et al., 2009).

A limited number of participants (3 in arm 1 and 5 in arm 2) reported musculoskeletal pain in the lower limb muscles following their engagement in aerobic activities during the initial week, which subsided during the third week of exposure. None of the subjects reported experiencing symptoms of angina or dyspnoea during the AE. Three individuals (two in arm 1 and one in arm 1) experienced non-syncopal falls during walking sessions and did not sustain major injuries. Seven participants experienced falls while ambulating during home exercises, and two participants sustained superficial bruises that resolved within 3 days. Two participants and one participant from arm 1 reported experiencing eye discomfort and mood disturbances following their involvement in the computerized cognitive training. All these problems diminished after the initial week. This indicates that the treatments were predominantly well accepted and feasible for older individuals with MCI, underscoring their potential for broader implementation in analogous populations.

The study's results have to be interpreted with caution. While widely acknowledged standards for change are lacking, a 5-point alteration in the modified mini-mental examination over a decade is deemed clinically significant in the older population (Andrew and Rockwood, 2008). The best type and dosage of exercise to enhance functionality in older individuals remain ambiguous. This study implemented home exercise interventions following a planned exercise regimen to improve recovery and general wellbeing. The home-based interventions were unsupervised, whereas center-based activities were supervised. The frequency and intensity of exercise prescriptions varied among these programs. Although participants completed the study, retention throughout its duration proved challenging, as they preferred remaining at home rather than attending outpatient therapy owing to transportation challenges and a lack of acclimatization to the environment specified for the exercises. Moreover, there is a deficiency of follow-up evidence concerning the potential enduring effects of exercise in elderly people, as well as the sustained maintenance of any benefits derived from exercise and modifications to health behaviors beyond the conclusion of a trial intervention. It is essential to assess the long-term efficacy and potential cost-effectiveness of home interventions; therefore, future research should investigate the sustained impact of both institution-based and home-based exercise programs.

The study had certain limitations. The reliance on self-reported data for certain measures may have introduced bias and affected the validity of the findings. Additionally, the psychological state of the participants was not explicitly assessed, which may have impacted the results. We did not categorize the subtypes of MCI or determine their predisposition to dementia. The absence of normative data on cortisol and cognitive function indicates that data from healthy elderly volunteers would have enhanced the results. No subgroup analysis was performed between elderly men and women, and previous studies have demonstrated inconsistent associations between sex and cortisol levels (Wolf et al., 2001; Karlamangla et al., 2005). Furthermore, the impact of education, smoking, comorbidities, prior physical activity, and smartphone usage was not collected and investigated. The utilization of objective measures to evaluate cognitive performance would provide more robust and reliable data, ultimately strengthening the evidence for these interventions. Conclusive cause-and-effect correlations between cognitive performance and cortisol could not be determined based on this result. Consequently, further investigation is required to evaluate the stress hypothesis by examining the impact of different dosages of exercise and cognitive interventions on the diurnal cortisol secretion pattern and various cognitive functions in individuals with MCI.

Considering the absence of recognized detrimental effects and the benefits of enhancing cognitive skills, intervention comprising cognitive training and AE may serve as a viable and well-tolerated method for older persons with MCI. It may be especially beneficial in situations where pharmacological interventions pose an increased risk of adverse consequences. Given the BI primarily measures physical independence not associated with cognitive-related functions, the utility of this daily living measure for assessing patients with MCI needs to be considered by future works.

## 5 Conclusion

The current study revealed that a sequential integration of aerobic and cognitive activities enhanced cognitive function and decreased cortisol levels at 16 weeks. Notwithstanding the intra-group enhancement, there was no statistically significant difference between the groups on the improvement of daily living tasks and quality of life. Nonetheless, additional research with bigger sample sizes is necessary to examine the elements, including varying exercise dosages, that affect the efficacy of the intervention, enabling individuals with MCI to take advantage of its benefits.

## Data Availability

The raw data supporting the conclusions of this article will be made available by the authors, without undue reservation.
